# The Nephroprotective Effects of α-Bisabolol in Cisplatin-Induced Acute Kidney Injury in Mice

**DOI:** 10.3390/biomedicines10040842

**Published:** 2022-04-03

**Authors:** Nur Elena Zaaba, Sumaya Beegam, Ozaz Elzaki, Javed Yasin, Bilal Mohamed Nemmar, Badreldin H. Ali, Ernest Adeghate, Abderrahim Nemmar

**Affiliations:** 1 Department of Physiology, College of Medicine and Health Sciences, United Arab Emirates University, Al Ain P.O. Box 17666, United Arab Emirates; elenazaaba@uaeu.ac.ae (N.E.Z.); sumayab@uaeu.ac.ae (S.B.); ozazelzaki@uaeu.ac.ae (O.E.); 2 Department of Internal Medicine, College of Medicine and Health Sciences, United Arab Emirates University, Al Ain P.O. Box 17666, United Arab Emirates; javed.yasin@uaeu.ac.ae; 3 College of Pharmacy, Gulf Medical University, Ajman P.O. Box 4184, United Arab Emirates; 2019ph19@mygmu.ac.ae; 4 Department of Pharmacology and Clinical Pharmacy, College of Medicine and Health Sciences, Sultan Qaboos University, Muscat P.O. Box 123, Oman; alibadreldin@hotmail.com; 5 Department of Anatomy, College of Medicine and Health Sciences, United Arab Emirates University, Al Ain P.O. Box 17666, United Arab Emirates; eadeghate@uaeu.ac.ae; 6 Zayed Center for Health Sciences, United Arab Emirates University, Al Ain P.O. Box 17666, United Arab Emirates

**Keywords:** cisplatin, nephrotoxicity, bisabolol, oxidative stress, inflammation

## Abstract

Cisplatin (CP) treatment has been long associated with the development of acute kidney injury (AKI) through mechanisms involving inflammation and oxidative stress. α-Bisabolol (BIS), a sesquiterpene alcohol isolated from the essential oil of various plants, including chamomile, has garnered popularity lately due to its antioxidant, anti-inflammatory, and anticancer properties. Therefore, we investigated the nephroprotective effects of BIS in the murine model of CP-induced AKI and the underlying mechanism of action. BALB/c mice were given BIS orally at 25 mg/kg for 7 days. On day 7, they were given a single dose of CP at 20 mg/kg intraperitoneally. BIS treatment continued for 3 more days. The animals were sacrificed at the end of the experiment (day 11). Kidneys, plasma, and urine were collected, and subsequently, various physiological, biochemical, and histological parameters were assessed. BIS has significantly normalized the alterations of water intake, urine volume, relative kidney weight, and the concentrations of urea and creatinine, as well as the creatinine clearance induced by CP treatment. BIS significantly mitigated the effects of CP-induced kidney injury by reducing kidney injury molecule-1, neutrophil gelatinase-associated lipocalin, adiponectin, and cystatin C. Likewise, the renal concentrations of proinflammatory cytokines, tumor necrosis factor α, interleukin (IL)-6 and IL-1β that were elevated in CP group were significantly reduced in mice treated with BIS and CP. A similar significant reduction was also observed in the CP-induced augmented levels of markers of oxidative stress, as well as the metabolite pteridine. Moreover, BIS significantly reduced the CP–induced renal DNA damage, and markedly lessened the acute tubular necrosis observed in kidney histology. Additionally, BIS significantly reduced the CP-induced increase in the phosphorylated nuclear factor κB (NFκB) in the kidney. These data strongly suggest that BIS exerts a protective action against CP-induced nephrotoxicity by mitigating inflammation and oxidative stress through the inhibition of NFκB activation. No overt adverse effects were noted with BIS treatment. Additional investigations should be done to consider BIS as an efficacious nephroprotective agent against CP.

## 1. Introduction

Cisplatin (CP) is one of the most effective and time-tested chemotherapeutic drugs, often used to treat a broad spectrum of solid malignancies such as testicular, cervical, ovarian, and lung cancers. Despite its efficacy and affordability, cisplatin has many harmful side effects, notably nephrotoxicity [1,2,3]. Acute kidney injury (AKI), a principal type of CP nephrotoxicity, is characterized by necrosis of the proximal tubule and collecting duct [4]. The mechanisms of CP-induced nephrotoxicity are multifactorial and include inflammation, oxidative stress, tubular cell injury, and apoptosis [2,5], leading to renal structural damage and an impairment in kidney function that is manifested in reduced glomerular filtration rate and several biochemical alterations that include the increase in the serum creatinine level, blood urea nitrogen (BUN), and several novel biomarkers [6,7,8]. 

CP-nephrotoxicity has limited the clinical use of CP in one in every three patients, and despite improvement in therapy, patients who developed AKI have an increased risk of death [9,10]. In spite of surviving AKI, the patient’s long-term prognosis is far from promising, with a substantial risk of progression to chronic kidney disease and end-stage renal disease [9,10].

Various drugs and supplements have been attempted to reverse or mitigate CP nephrotoxicity. These include antioxidants (e.g., melatonin and vitamin E) [11], modulators of nitric oxide (e.g., zinc histidine complex) [12], diuretics (e.g., furosemide and mannitol) [13], cytoprotective and anti-apoptotic agents (e.g., amifostine and erythropoietin) [14]. However, none of the data have shown complete nephroprotection in both human and animal models [15].

Bisabolol (BIS), formally known as α-(−)-bisabolol, is an oily sesquiterpene alcohol derived from various types of plants, primarily from German chamomile (*Matricaria chamomilla*). It has been traditionally used in the production of cosmetics and perfumes and has gained a lot of attention in recent years due to its cytotoxic [16], anticholinesterasic [17], antitumorigenic [18], anti-inflammatory [19], and antioxidant properties [20] in rodent models.

As CP exerts inflammatory and oxidative actions, and BIS has antioxidant and anti-inflammatory activities [21,22], we thought it would be interesting to evaluate whether BIS could mitigate or prevent CP nephrotoxicity in mice. To the best of our knowledge, such a study has not been reported before. Therefore, this study aims to assess the possible ameliorative or protective effects of BIS on CP-induced nephrotoxicity and the underlying mechanism of action.

## 2. Materials and Methods

### 2.1. Animals

An equal number of male and female BALB/c mice (Taconic Farms Inc., Germantown, NY, USA) weighing 25–30 g were housed in a conventional animal house and maintained on a 12-h light–dark cycle with a relative humidity of 50–60% and temperature-controlled (22 ± 1 °C) rooms. They were supplied with commercial additive-free laboratory chow consisting of 24% crude protein, 2% crude fat, and 8% crude fiber (National Feed and Flour and Marketing Co., Abu Dhabi, United Arab Emirates) and drinking water ad libitum. 

### 2.2. Experimental Design

The mice were randomly segregated into four groups (n = 8), with two groups receiving 25 mg/kg BIS bought from Sigma Aldrich Co. (St. Louis, MO, USA) [23,24].

Group 1. Control-received sunflower oil (10 mL/kg) via oral gavage for 10 days and saline (10 mL/kg) intraperitoneal (IP) injection was administered on day 7.

Group 2. CP-received sunflower oil (10 mL/kg) via oral gavage for 10 days and an IP injection of CP (20 mg/kg) was given on day 7.

Group 3. BIS-received BIS (25 mg/kg, dissolved in sunflower oil) via oral gavage for 10 days, and was given saline (10 mL/kg) IP injection on day 7.

Group 4. BIS + CP–received BIS (25 mg/kg, dissolved in sunflower oil) via oral gavage for 10 days and was given an IP injection of CP (20 mg/kg) on day 7.

**Figure 1:**
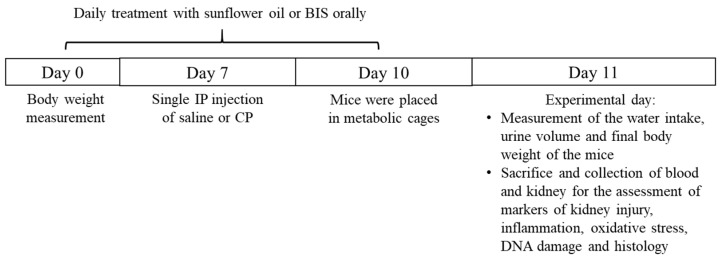
The treatments and endpoints following daily oral treatment of either sunflower oil or bisabolol (BIS, 25 mg/kg) with or without cisplatin (CP, 20 mg/kg) given intraperitoneally (IP).

The animals were sacrificed on day 11 of the experiment (Figure 1), and 24 h prior to sacrifice, they were housed individually in metabolic cages for urine collection. The volume of urine voided and water intake during the 24 h were recorded, and collected urine was stored at −80 °C. 

The bodyweight of the mice was recorded at the beginning of the study and right before sacrifice. On the day of the sacrifice, the mice were anesthetized with a dose of (60 mg/kg, IP) sodium pentobarbital. The blood was obtained via the inferior vena cava and centrifuged at 4 °C for 15 min at 900× *g*. The plasma samples were then stored at −80 °C pending analysis. The kidneys were excised and weighed. A small portion of the top part of the right kidney was excised and fixed in 10% formalin for histopathological analysis. The remainders of both right and left kidneys were rapidly wrapped and snap-frozen in liquid nitrogen and kept at −80 °C to await biochemical analysis.

### 2.3. Homogenization of Kidney

Frozen kidney tissues were thawed and transferred into sterile 2-mL homogenization microvial along with 2.0 mm Zirconia beads from BioSpec (Bartlesville, OK, USA), and homogenized in KCl buffer supplemented with protease and phosphatase inhibitor cocktail using the Precellys homogenizer from Bertin Instrument (Bretonneux, France) for five times of 3 cycles of 10 s at 6500× *g*. Homogenates were centrifuged for 20 min at 14,000× *g* to remove cellular debris. The supernatants were divided into aliquots and kept at −80 °C pending analysis. 

The protein content of kidney homogenate was measured as per the protocol in the Pierce™ BCA Protein Assay Kit (Thermo Scientific, Rockford, IL, USA). 

### 2.4. Biochemical Analysis

The concentrations of creatinine and urea were measured using commercial kits from Roche Diagnostics (Indianapolis, IN, USA).

### 2.5. Measurement of Markers of Inflammation, Kidney Injury and Oxidative Stress in Kidney Homogenates, Plasma, and Urine

The concentrations of the tumor necrosis factor α (TNFα), interleukin (IL)-6 and IL-1β in kidney homogenates and the kidney injury markers, kidney injury molecule-1 (KIM-1), neutrophil gelatinase-associated lipocalin (NGAL), adiponectin, and cystatic C in plasma were quantified using commercially available ELISA kits manufactured by R&D Systems (Minneapolis, MN, USA). The marker of proximal tubular damage N-acetyl-β-glucosamide (NAG) in urine, on the other hand, was measured using an assay kit from MyBiosource (San Diego, CA, USA). The concentrations of superoxide dismutase (SOD) in kidney homogenates and 8-hydroxy-2′-Deoxyguanosine (8-OH-dG) in urine were determined as per the protocols that came with the ELISA kits from Cayman Chemicals (Ann Arbor, MI, USA).

### 2.6. Measurement of Thiobarbituric Acid Reactive Substances (TBARS) and Reduced Glutathione (GSH) in Kidney Homogenates

The lipid peroxidation (LPO) level was estimated spectrophotometrically using the thiobarbituric acid reactive substances (TBARS) method and the malondialdehyde (MDA) standard curve [25,26,27]. Meanwhile, the Ellman’s reagent (5,5′-dithio bis2-nitrobenzoic acid) and the GSH calibration curve were used to quantify the non-enzymatic antioxidant level of reduced glutathione (GSH) as described in the previous publication [28]. The yellow-colored complex produced was measured at 412 nm.

### 2.7. Assessment of Pteridine in Urine

The concentration of neopterin, the oxidized form of pteridine derivatives, were assessed using a method previously described [29]. The standard curve for the measurement of pteridine was generated using neopterin at the concentrations of 0, 6.25, 12.5, 25, 50, and 100 μM. The urine samples were diluted with 10 mM HEPES buffer (pH 7.0) at 1:50 prior to the experiment and reading was done with an excitation wavelength of 360 nm and an emission wavelength of 450 nm. 

### 2.8. DNA Damage Assessment by Comet Assay

In a separate set of mice (n = 5), the kidneys were excised promptly after sacrifice and processed to assess the DNA damage using the COMET assay [30,31,32]. The assessment of the length of DNA migration was measured using image analysis Axiovision 3.1 software (Carl Zeiss, Toronto, ON, Canada).

### 2.9. Measurement of Phosphorylated NF-κB in Kidney Homogenates

The measurement of the phosphorylated NF-κB (Cell Signaling Technology, Danvers, MA, USA) in kidney homogenates was done using a commercially available ELISA kit. 

### 2.10. Histopathology

Following the standard histopathological protocol, the formalin-fixed tissue samples were embedded in paraffin wax, then they were cut into 5 µm thickness slices using a microtome (RM2125 RTS, Leica Biosystems, Nussloch, Germany) [33,34]. The sections were then mounted on slides and stained with hematoxylin and eosin. The stained sections were evaluated and scored randomly by the histologist using light microscopy. Scoring was done based on the percentage of acute renal tubular necrosis using a semi-quantitative method. Normal kidney architecture, which exhibited no necrosis, was given 0 points, less than 10% necrosis was given 1 point, 2 points for 10–25%, 3 points for 26–75%, and 4 points for tissues that exhibited more than 75% of necrosis [33,34].

### 2.11. Statistics

All data generated from this study were analyzed using the GraphPad Prism Software 7 (San Diego, CA, USA). The one-way analysis of variance followed by Holm–Sidak’s multiple comparison test was used. Data in the figures of this study were reported as mean ± SEM. *p* values < 0.05 are considered statistically significant.

## 3. Results

### 3.1. The Effects of BIS on Physiological and Biochemical Parameters

**Figure 2:**
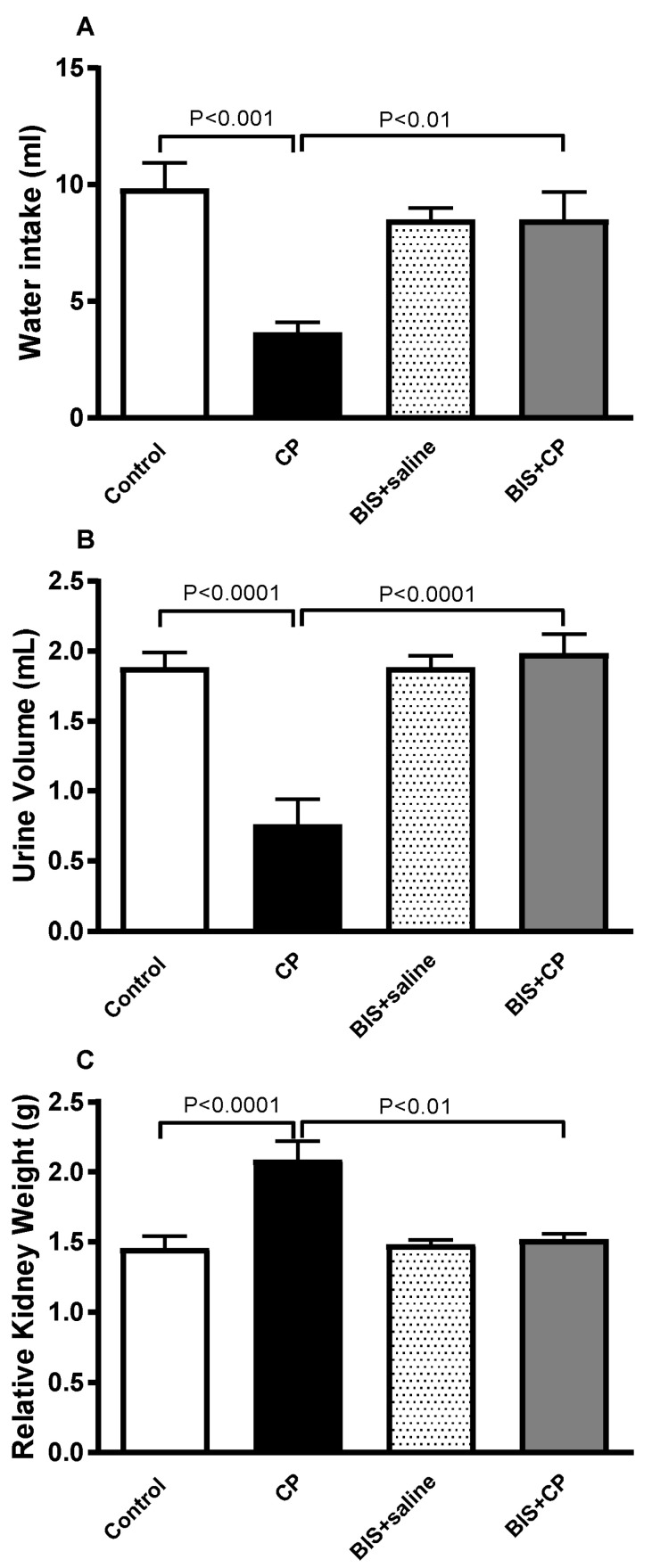
Water intake (**A**), urine volume (**B**), and relative kidney weight (**C**) in mice treated orally with either sunflower oil (control) or bisabolol (BIS, 25 mg/kg) with or without cisplatin (CP, 20 mg/kg) given intraperitoneally (n = 6). Mean ± SEM. Statistical analysis by ANOVA 1 followed by Holm Sidak’s test.

Figure 2 shows that there was a significant reduction in water intake (*p* < 0.001) and urine volume (*p* < 0.0001) in the CP group. Conversely, the relative kidney weight was significantly increased in the CP group compared to the control (*p* < 0.0001). A significant protective effect was observed when treatment with CP is given along with BIS for water intake (*p* < 0.01), urine volume (*p* < 0.0001), and relative kidney weight (*p* < 0.01).

**Figure 3:**
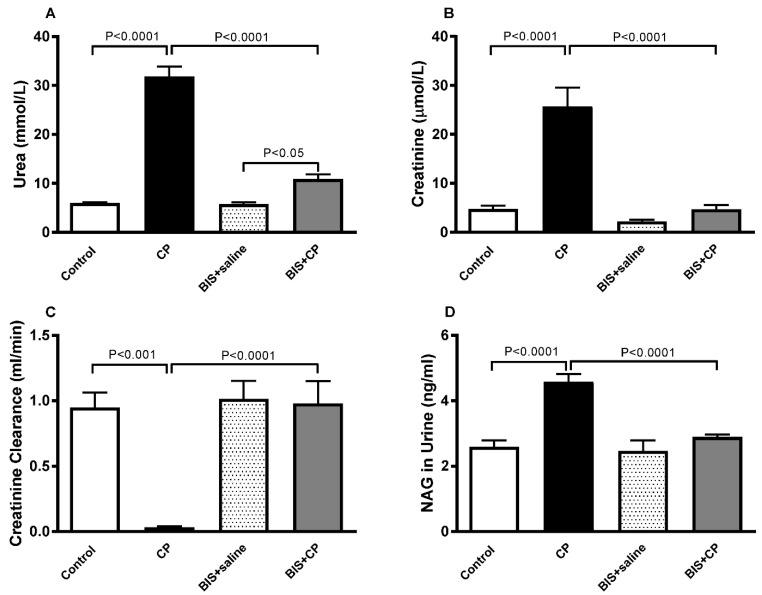
The plasma levels of urea (**A**) and creatinine (**B**), and creatinine clearance (**C**), as well as the level of urine NAG (**D**) in mice treated orally with either sunflower oil (control) or bisabolol (BIS, 25 mg/kg) with or without cisplatin (CP, 20 mg/kg) given intraperitoneally (n = 6). Mean ± SEM. Statistical analysis by ANOVA 1 followed by Holm–Sidak’s test.

Our biochemical analysis has shown a significant surge in plasma urea (*p* < 0.0001) and creatinine (*p* < 0.0001) in the CP group compared with the control. However, when CP was given together with BIS, the levels of these analytes were significantly diminished compared with the CP group (*p* < 0.0001 and *p* < 0.0001; Figure 3A,B). CP caused a significant decrease in creatinine clearance (*p* < 0.001), and a significant increase was observed in the BIS + CP group when compared with the CP-treated animals (*p* < 0.0001; Figure 3C). Figure 3D shows that CP caused a significant elevation in the activity of NAG (*p* < 0.0001), a marker for proximal tubule damage, and an opposite effect was observed when the CP-treated mice were given BIS (*p* < 0.0001).

### 3.2. Effects of BIS on NGAL, KIM-1, Cystatin C and Adiponectin in Plasma 

**Figure 4:**
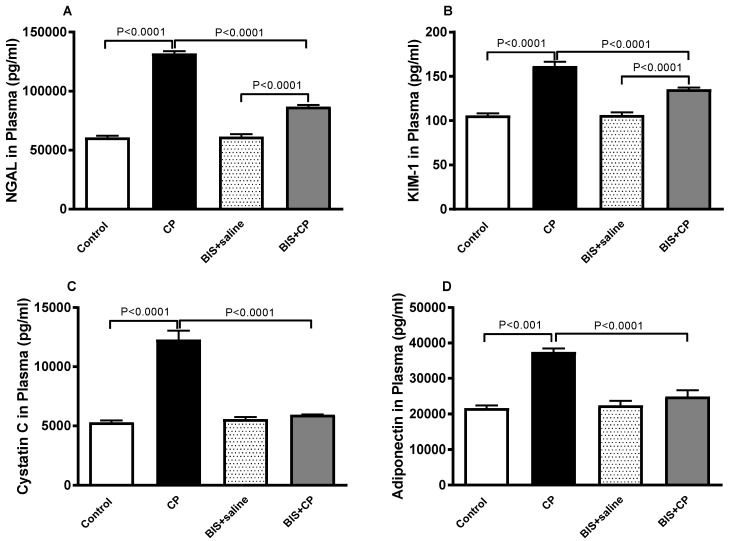
Plasma levels of neutrophil gelatinase-associated lipocalin (NGAL, (**A**)), kidney injury molecule-1 (KIM-1, (**B**)), cystatin C (**C**), and adiponectin (**D**) in mice treated orally with either sunflower oil (control) or bisabolol (BIS, 25 mg/kg) with or without cisplatin (CP, 20 mg/kg) given intraperitoneally (n = 8). Mean ± SEM. Statistical analysis by ANOVA 1 followed by Holm–Sidak’s test.

Figure 4 depicts the effect of BIS on biomarkers of kidney injury and renal dysfunction. CP treatment has caused a significant surge in NGAL and KIM-1 (*p* < 0.0001 and *p* < 0.0001) in plasma. 

When treatment of BIS was given in parallel with CP, the plasma concentration of NGAL and KIM in the BIS + CP group exhibited significant reduction (*p* < 0.0001 and *p* < 0.0001) compared with the CP alone group. 

CP caused significant increases in the concentrations of cystatin C and adiponectin (*p* < 0.0001 and *p* < 0.001, respectively). These effects were significantly reversed when CP was given concomitantly with BIS.

### 3.3. Effects of BIS on the Concentrations of Pro-Inflammatory Cytokines in Kidney Homogenate

**Figure 5:**
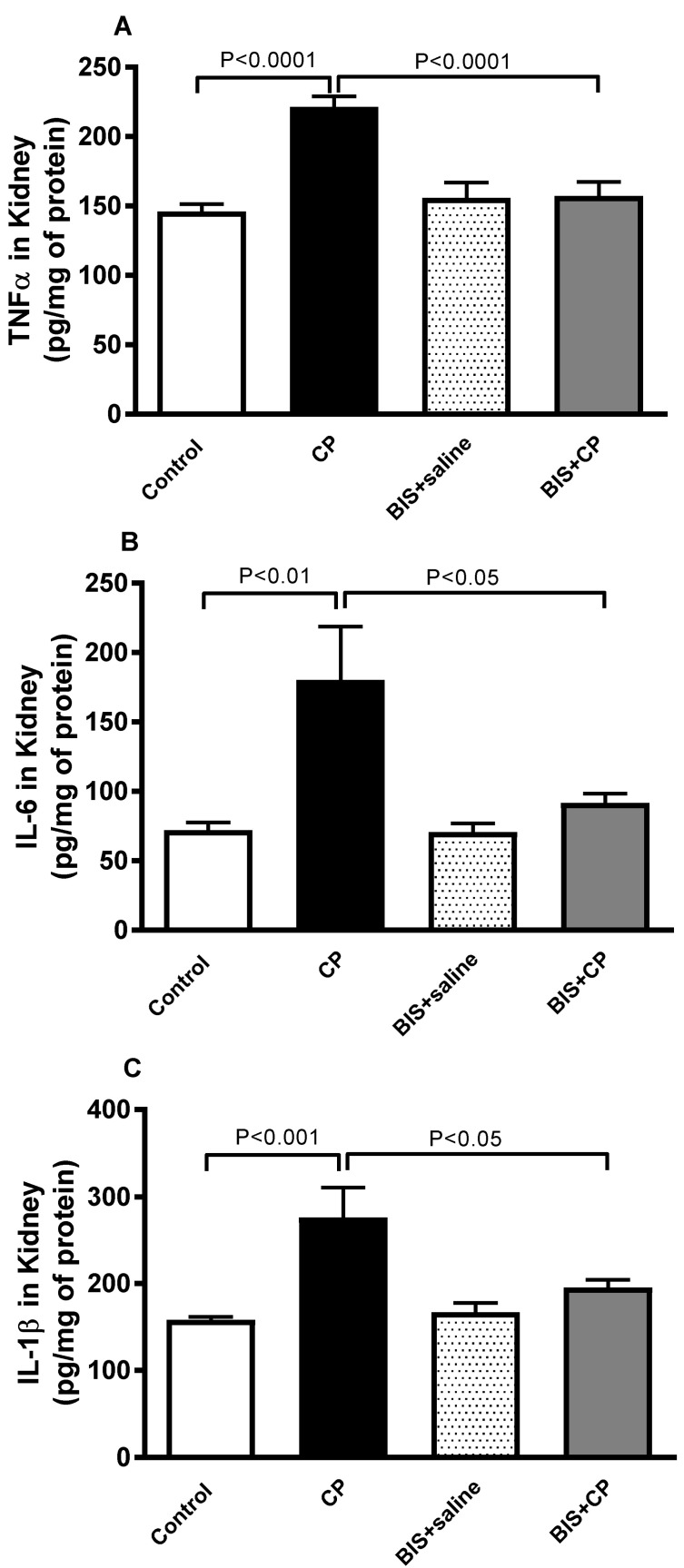
The renal concentrations of tumor necrosis factor-alpha (TNF α (**A**)), interleukin (IL)-6 (**B**), and IL-1β (**C**) in mice treated orally with either sunflower oil (control) or bisabolol (BIS, 25 mg/kg) with or without cisplatin (CP, 20 mg/kg) given intraperitoneally (n = 7–8). Mean ± SEM. Statistical analysis by ANOVA 1 followed by Holm–Sidak’s test.

The concentrations of markers of inflammation TNFα, IL6, and IL1β in kidney were significantly elevated in CP treated mice (*p* < 0.0001, *p* < 0.01, and *p* < 0.001, respectively) when compared with the control group. These elevations were significantly diminished in mice treated with both BIS and CP (*p* < 0.0001, *p* < 0.05, and *p* < 0.05, respectively) when compared with mice treated with CP alone (Figure 5).

### 3.4. Antioxidant Effects of BIS in Kidney

**Figure 6:**
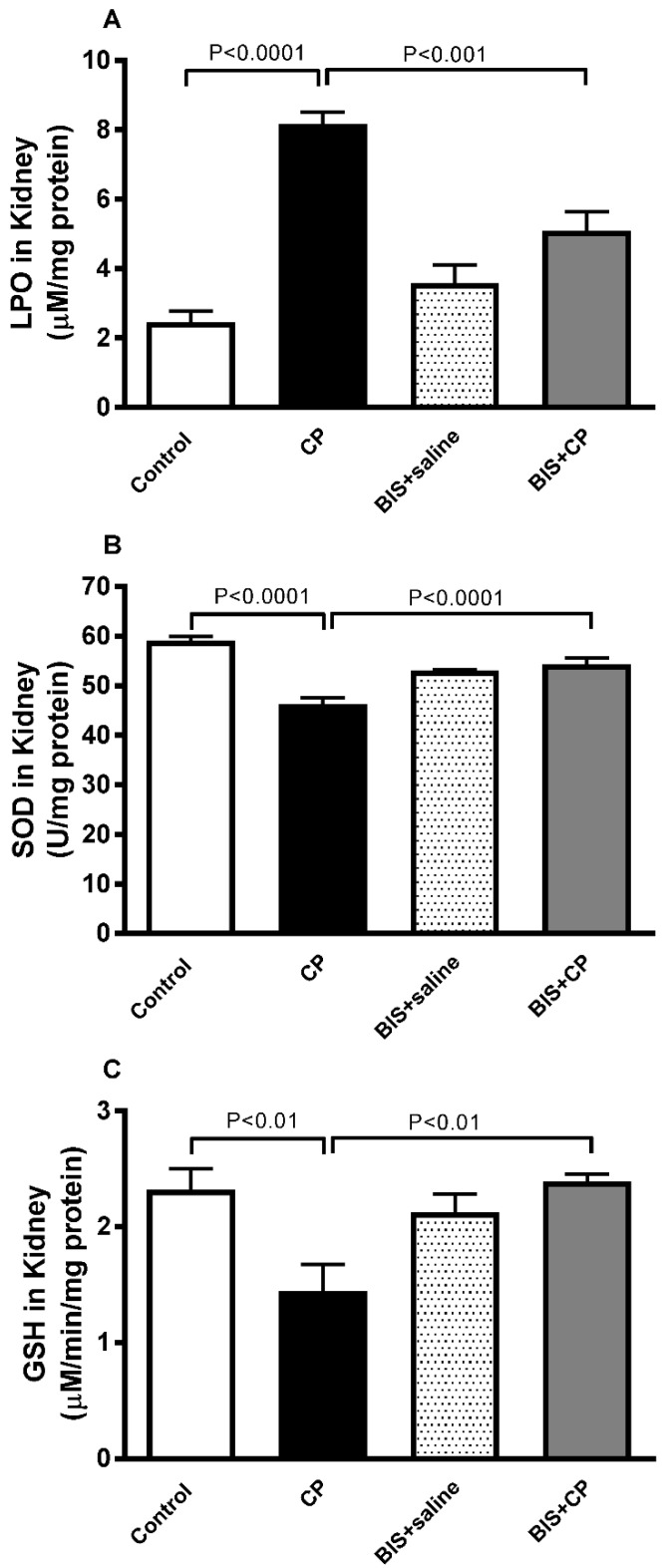
The renal levels of lipid peroxidation (LPO, (**A**)), superoxide dismutase (SOD, (**B**)), and reduced glutathione (GSH, (**C**)) in mice treated orally with either sunflower oil (control) or bisabolol (BIS, 25 mg/kg) with or without cisplatin (CP, 20 mg/kg) given intraperitoneally (n = 8). Mean ± SEM. Statistical analysis by ANOVA 1 followed by Holm–Sidak’s test.

CP induced a significant elevation in the level of LPO (*p* < 0.0001). The latter effect was markedly and significantly reduced in BIS + CP group compared with the CP group (*p* < 0.001; Figure 6A). In contrast, a significant reduction was observed in SOD activity and the concentration of GSH (*p* < 0.0001 and *p* < 0.01, respectively) in CP-treated mice compared to control. These reductions were significantly reversed (*p* < 0.0001 and *p* < 0.01) when CP was administered simultaneously with BIS (Figure 6B,C).

### 3.5. Effects of BIS on Kidney DNA Damage

**Figure 7:**
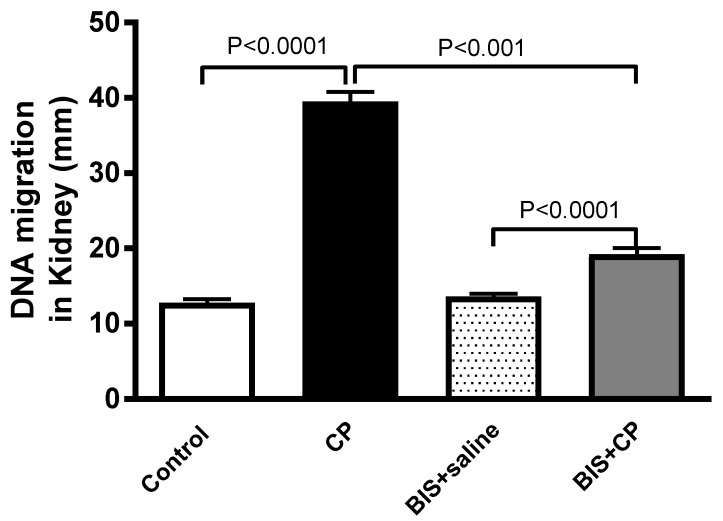
DNA migration (mm) in kidney tissue assessed by Comet assay in mice treated orally with either sunflower oil (control) or bisabolol (BIS, 25 mg/kg) with or without cisplatin (CP, 20 mg/kg) given intraperitoneally (n = 5). Mean ± SEM. Statistical analysis by ANOVA 1 followed by Holm Sidak’s test.

Figure 7 depicts the evaluation of DNA damage in the kidney tissue. The CP group exhibited a significant increase in DNA damage compared with the control group (*p* < 0.0001). However, when BIS was given concomitantly with CP, a significant mitigating effect was observed (*p* < 0.001).

### 3.6. Effects of BIS on 8-OH-dG and Pteridine Concentrations in Urine

**Figure 8:**
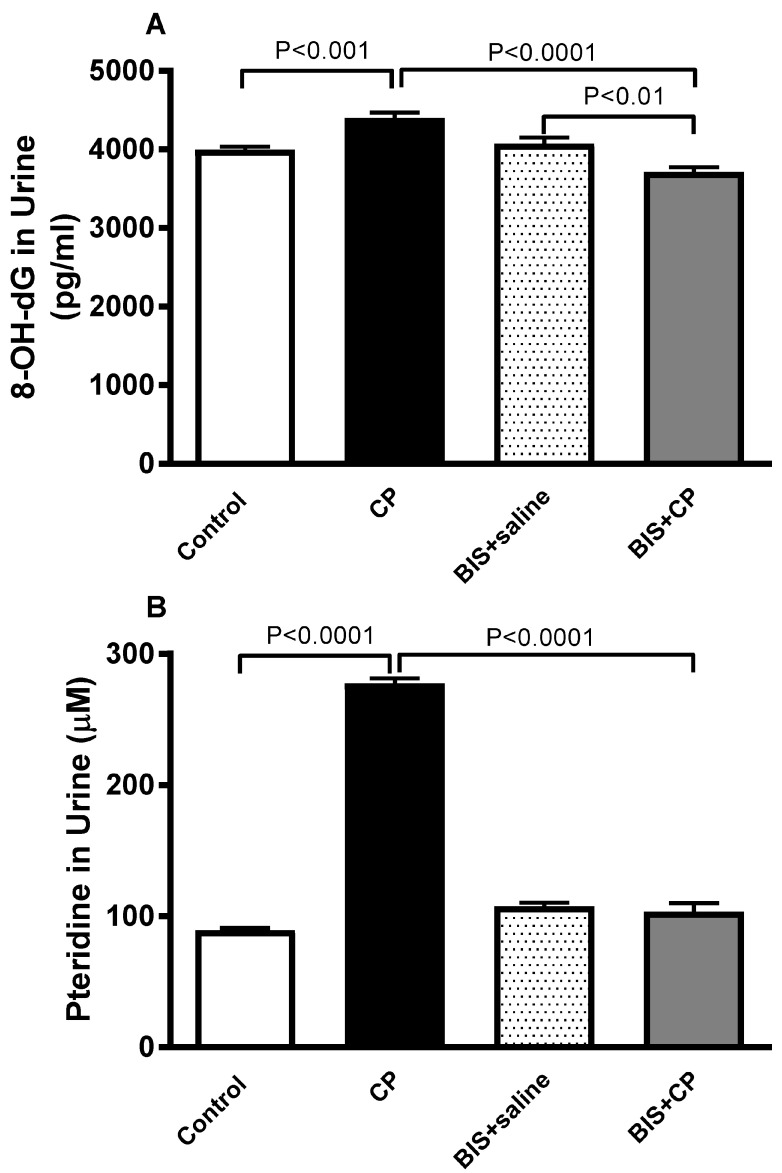
Urine levels of 8-hydroxy-2-deoxyguanosine (8-OH-dG, (**A**)) and pteridine (**B**) in mice treated orally with either sunflower oil (control) or bisabolol (BIS, 25 mg/kg) with or without cisplatin (CP, 20 mg/kg) given intraperitoneally (n = 6). Mean ± SEM. Statistical analysis by ANOVA 1 followed by Holm–Sidak’s test.

The concentration of 8-OH-dG (Figure 8A), a marker of oxidative DNA damage in urine, was significantly augmented in the CP group (*p* < 0.001) compared to the control. This effect was significantly normalized when CP was given simultaneously with BIS (*p* < 0.0001). The level of Pteridine was significantly increased in CP treated group compared to the control mice (*p* < 0.0001). Nevertheless, this effect was significantly reversed in BIS + CP group compared to mice treated with CP alone (*p* < 0.0001; Figure 8B).

### 3.7. Effects of BIS of the Expression of Phopho-NF-κB in Kidney Homogenate

**Figure 9:**
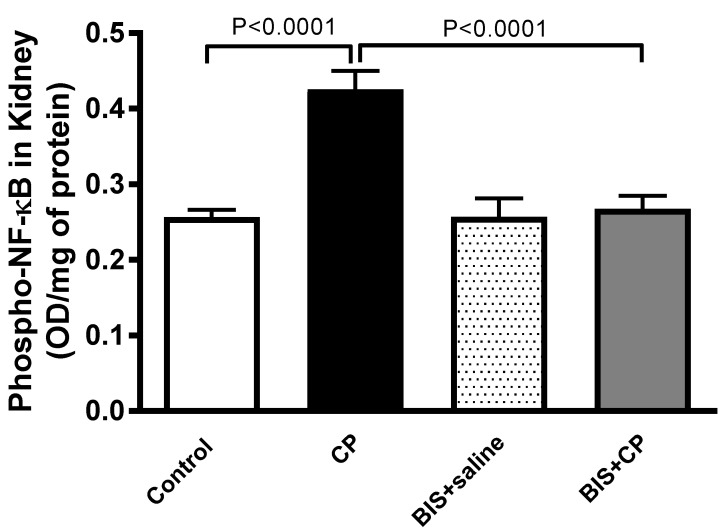
Phosphorylated nuclear factor-κB (phospho-NF-κB) expression in the kidney of mice treated orally with either sunflower oil (control) or bisabolol (BIS, 25 mg/kg) with or without cisplatin (CP, 20 mg/kg) given intraperitoneally (n = 8). Mean ± SEM. Statistical analysis by ANOVA 1 followed by Holm Sidak’s test.

Figure 9 shows that the levels of phopho-NF-κB in the CP group were significantly augmented compared to the control group (*p* < 0.0001). The latter effect was completely abrogated by BIS treatment (*p* < 0.0001).

### 3.8. Effects of BIS on Kidney Histology Assessed by Light Microscopy 

**Figure 10:**
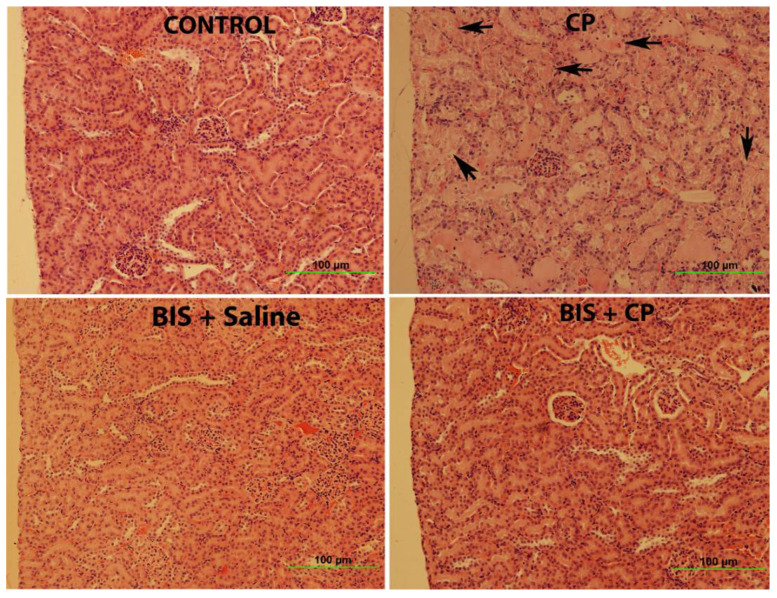
Representative light microscopy sections of renal tissue of mice treated orally with either sunflower oil (control) or bisabolol (BIS, 25 mg/kg) with or without cisplatin (CP, 20 mg/kg) given intraperitoneally (n = 6). The kidney architecture was not affected in the control and BIS-treated groups, with the renal tubules showing a normal appearance and scoring 0. The image in the CP group showed acute tubular necrosis (arrows) was given the score of 3, while the BIS + CP group showed significant improvement in the histological appearance compared to the CP group was scored 1.

Figure 10 and Table 1 illustrate the results of renal histopathology examinations. Histological evaluation and scoring showed that the control group displayed normal kidney architecture with a score of 0. The kidney section from the CP-treated animals was scored 3 as they exhibited acute tubular necrosis at 58% of the examined tissue area with tubular dilatation, interstitial edema, and congestion. The BIS-treated group showed normal kidney architecture and histology and scored 0. The BIS + CP treated group showed significant improvement in the histological appearance compared to the CP group and was scored 1. 

## 4. Discussion

In this study, we show that the treatment with a daily dose of 25 mg/kg BIS has significantly abrogated the biochemical, histopathological, and molecular changes, including inflammation, kidney injury, oxidative stress, and DNA damage, induced by CP in the kidney, without causing any overt adverse side effects. 

BIS is a non-toxic plant derivative with an oral LD_50_ of 13–14 g/kg of bodyweight in rodents [35,36] that, when administrated on its own at 25 mg/kg, did not cause any adverse effect and did not change any parameters evaluated in this study. The dosage has been selected based on published studies showing its efficacy in the attenuation of inflammation and oxidative stress in rodent models of nociception, myocardial infarction and neuronal damage [19,37,38,39].

Clinical and experimental studies have demonstrated that CP treatment alters both physiological and biochemical parameters in patients and animal models [1,15]. In the present study, CP treatment has caused physiological changes, including a significant reduction in water intake, urine volume, and an increase in relative kidney weight. A similar observation is found in published CP-induced nephrotoxicity studies in mice [40,41]. The decrease in water intake may be due to the gastrointestinal toxicity inflicted by CP [42], which resulted in modified eating and drinking habits. This, in turn, led to the reduction of urine output. The increase in relative kidney weight may be due to hypertrophy, an adaptive growth of kidney tissue to restore the lost renal function [5].

The plasma creatinine, urea, and creatinine clearance are standard biochemical indices for nephrotoxicity [8]. These indices were significantly altered by CP and normalized with the treatment of BIS.

In the present work, we have also included the assessment of various novel kidney injury biomarkers to support our findings, such as NAG, NGAL, KIM-1, cystatin C, and adiponectin. These sensitive biomarkers are reliably used in diagnosing nephrotoxicity and have been applied in previous acute kidney injury studies [40,43]. Our results have shown a significant increase in the levels of NAG, NGAL, KIM-1, cystatin C, and adiponectin in CP-treated mice, and treatment with BIS has demonstrated the restoration to these kidney injury biomarkers.

Inflammation and oxidative stress are interconnected mechanisms that modulate CP-induced nephrotoxicity. CP accumulation triggers the signaling cascade that leads to the onset of inflammation, which subsequently induces the activation of TNF α, which in turn promotes the secretion of other cytokines, including IL-6 and IL-1β [44,45,46]. Concurrently, the overproduction of reactive oxygen species (ROS) at the site of inflammation plays an important role in the induction of oxidative stress [47]. In the present study, we have demonstrated that the levels of TNF α, IL-6, and IL-1β were significantly increased in the kidneys of the CP-treated mice, and the treatment with BIS has markedly altered the increase. Furthermore, the oxidative stress due to CP induction increases the event of lipid peroxidation where the polyunsaturated fatty acids of cell membranes are cleaved at their double bonds, resulting in the formation of aldehydes, MDA, and 4-hydroxynonenal [48]. In this study, we have measured the level of TBARS, a customary marker used to quantify lipid peroxidation [49]. CP treatment has significantly increased the lipid peroxidation, which corroborates with previous findings [44,50] and BIS at the dose used was found to be effective in abrogating this altered effect.

The overproduction of ROS leads to the reduced production of the antioxidants SOD and GSH [51], a finding observed in our study. The reduction of these antioxidants may in part be due to their consumption during the breakdown of free radicals [52], an event that was significantly and effectively reversed by BIS. 

It has been reported that the increase in ROS and the binding of CP with DNA to form covalent platinum DNA adducts result in DNA damage [53,54]. Therefore, we have assessed the DNA damage using Comet assay in kidney tissue in the present study. Our data show that CP markedly induced DNA injury. A significant reduction in DNA damage activity was observed in mice with CP-induced nephrotoxicity treated with BIS, as opposed to the CP-only group. In addition to assessing DNA damage by Comet assay, we have measured the accumulation of a biomarker of DNA and RNA oxidative damage, 8-OH-dG in urine. 8-OH-dG, one of the principal forms of free radical-induced oxidatively-modified DNA, is water-soluble and excretable via urine [55]. It is a standard measurement to evaluate the extent of DNA damage of various conditions, especially AKI, due to its non-invasiveness, its proximity to the injury, and as a site of the accumulation of biomarkers released by damaged kidney tissues [56,57]. Here, we found that CP treatment has significantly elevated the level of 8-OH-dG, an action that was significantly reduced by the treatment of BIS. 

In this study, we have demonstrated, probably for the first time, the effect of CP treatment on the concentration of urinary pteridine, an important modulator of oxidative stress [58]. Neopterin, the oxidized form of pteridine, has been used to measure the level of oxidative stress in patients with cancer [59], cardiovascular [60], and infectious diseases [61,62]. This fluorescence emitting pteridine derivative is detectable by spectrofluorometry as opposed to its reduced form. Here, we have observed that CP treatment has significantly elevated the level of pteridine, an action that was significantly reversed by the treatment of BIS. 

To further explore the mechanisms underlying the ameliorative effects of BIS on CP-induced oxidative stress and inflammation, we assessed the NF-κB expression in the kidney. NF-κB is well-known to be implied in the pathophysiology of various inflammatory diseases, in particular those affecting the kidney, as it plays a significant role in actuating the transcription of inflammatory cytokines, leading to inflammation and oxidative stress [63]. Our data demonstrated that BIS administration prevented the production of inflammatory cytokines including IL-6, TNFα and IL-1β, and NF-κB in the kidney. This effect may be ascribed to the anti-inflammatory action of BIS in the kidney tissue. Our findings corroborate a recent study that showed that BIS administration suppresses the inflammatory response and extracellular matrix catabolism in advanced glycation end products-treated chondrocytes and attenuates murine osteoarthritis by blocking NF-κB activation [64].

Histologically, CP has caused significant structural damage in the kidney. This is in line with the findings that demonstrated CP-induced nephrotoxicity in a similar animal model [40,50,65]. Treatment with BIS has attenuated the damage observed in CP treated group. This protective effect may be due to the reduction of CP-related inflammation, oxidative stress, and DNA damage.

## 5. Conclusions

In conclusion, our data strongly suggest that BIS exerts a protective action against CP-induced nephrotoxicity by mitigating inflammation and oxidative stress through the inhibition of NFκB activation. No overt adverse effects were noted with BIS treatment. Additional investigations should be done to consider BIS as an efficacious nephroprotective agent against CP. 

## Figures and Tables

**Table 1:** Histopathological analysis and scoring of renal sections of mice treated orally with either sunflower oil (control) or bisabolol (BIS, 25 mg/kg) with or without cisplatin (CP, 20 mg/kg) given intraperitoneally.

Group	% of Necrotic Area	Necrosis Score
Control	0	0
CP-treated	58 ± 3.0 ****	3
BIS-treated	0	0
BIS and CP-treated	2 ± 0.6 ^ΔΔΔΔ^	1

Data are expressed as mean ± SEM (n = 6). Statistical analysis by ANOVA 1 followed by Holm–Sidak’s test. **** *p* < 0.0001 compared with the control group and ^ΔΔΔΔ^ *p* < 0.0001 compared with the CP-treated group.

## Data Availability

The data presented in this study are available on request from the corresponding author.
